# Bond order redefinition needed to reduce inherent noise in molecular dynamics simulations

**DOI:** 10.1038/s41598-020-80217-0

**Published:** 2021-02-11

**Authors:** Ibnu Syuhada, Nikodemus Umbu Janga Hauwali, Ahmad Rosikhin, Euis Sustini, Fatimah Arofiati Noor, Toto Winata

**Affiliations:** Physics of Electronic Materials Research Division, Department of Physics, Faculty of Mathematics and Natural Sciences, Institut Teknologi Bandung, Jalan Ganesha 10, Bandung, 40132 Indonesia

**Keywords:** Atomic and molecular physics, Condensed-matter physics, Condensed-matter physics

## Abstract

In this work, we present the bond order redefinition needed to reduce the inherent noise in order to enhance the accuracy of molecular dynamics simulations. We propose defining the bond order as a fraction of energy distribution. It happens due to the character of the material in nature, which tries to maintain its environment. To show the necessity, we developed a factory empirical interatomic potential (FEIP) for carbon that implements the redefinition with a short-range interaction approach. FEIP has been shown to enhance the accuracy of the calculation of lattice constants, cohesive energy, elastic properties, and phonons compared to experimental data, and can even be compared to other potentials with the long-range interaction approach. The enhancements due to FEIP can reduce the inherent noise, then provide a better prediction of the energy based on the behaviour of the atomic environment. FEIP can also transform simple two-body interactions into many-body interactions, which is useful for enhancing accuracy. Due to implementing the bond order redefinition, FEIP offers faster calculations than other complex interatomic potentials.

## Introduction

In atomic simulations, there are several methods often used to describe the interaction of atoms such as density functional theory (DFT)^1,2^, density-functional tight-binding (DFTB)^3,4^, and molecular dynamics (MD)^5–8^. Of those methods, DFT has higher accuracy compared to experiments than the others. Due to the complexity of its calculations, however, DFT is only efficient to use for hundreds of atoms and to model several femtoseconds, making DFT is an expensive method due to the dependency on the use of a supercomputer. The DFT approximation, i.e. DFTB, which works based on the second-order expansion of Kohn–Sham energy, where the Hamiltonian elements and the overlap matrix are determined via a parameterising procedure, can simulate up to hundreds of picoseconds 1000 times faster than DFT. In practice, however, this method still not efficient for large masses of atoms. Thus, MD is often used as a solution for studying atomic mechanisms.

In the MD simulation, the interatomic potential plays a role in describing the interactions between the atoms. The quality of the interatomic potential determines the accuracy of the simulation compared to the experiments. Attempts to improve the quality by including bond order functions have resulted in the short-range empirical interatomic potential (EIP)^9–11^; the EIP is a specific case for atoms interaction with the first nearest neighbours. Furthermore, there is the analytical interatomic potential, i.e. the analytic bond order potential (ABOP)^12–15^. The principle in ABOP is that the bond order is the difference between the bonding and antibonding electron states. Based on this approach, the bond order definition adopted by this potential offers better replication of atomic phenomenon. Iron screw dislocation is one interesting study undertaken using this potential^16,17^. However, for the body-centred cubic (BCC) structure of iron, the kink-pair nucleation process is still not reproduced well by simulations. In addition, even though it has successfully modelled many things, the MD simulation with ABOP potential remains an expensive calculation to simulate the complex plasticity phenomenon.

Associated with the complexity calculation of ABOP, an approach which still takes into account the influence of long-range interactions is the “long-range” carbon bond order potential (LCBOP)^18^. It has the accuracy to describe phenomena which relate to the carbon, particularly with regard to the atomic dynamics process of diamond graphitisation. However, this potential applies only to carbon–carbon interactions. The latest promising potential development for improving MD is machine learning (ML)-based potentials^19–21^. The purpose of ML-based potentials is to produce computations with quantum mechanical (QM) accuracy but with a lower computational cost than DFT. The strategy is to maps a set of atomic environments directly into numerical values (as a fingerprint) for energies and forces. Thus, the success of this method lies in the accuracy of the fingerprint selection. In addition, the atomic environment structure database references are accumulated via separate ab-initio computations and nudged elastic bands (NEB)^22–24^. A database training process is then adopted to establish the mapping between fingerprints and atomic energies, and the output is the interatomic potential. Therefore, the ML method still has a computationally high cost to simulate large numbers of atoms.

Furthermore, Pauling showed experimentally that the bond order has a close relation to the bond length^25^. The latest technique is density derived electrostatic and chemical (DDEC6), which has been reported as the best method for calculating the bond order^26^. This method applies to non-magnetic, collinear magnetic and non-collinear magnetic materials with localised or delocalised bonding electrons. Even so, DDEC6 is not designed for electrodes, highly time-dependent states, some extremely high-energy excited states, and nuclear reactions. However, no absolute definition for the bond order, but we can indicate its quantity via bond length, coordination number, bond angle, dihedral, and charge populations. Regarding MD simulation, though the ML method offers QM accuracy, in general, the simulation calculation depends on the underlying potential, especially the definition of bond order. In particular, there is inherent noise in the MD simulation, which affects the results.

In this study, we show the need for bond order redefinition to reduce inherent noise in MD simulations. Through this redefinition, the beginning of a factory-based empirical interatomic potential (FEIP) is developed in this study. Thus, for a simple potential such as Lennard–Jones, the accuracy can increase when it is transformed into a many-body potential. We use the carbon–carbon interaction for graphene phonons dispersion, mechanical properties and the cohesive energy of graphite and diamond, to show how this redefinition affects the accuracy and the speed of the simulation, which is useful for future studies of atomic mechanisms.

## Results

### Bond order redefinition

We begin with the principles of the redefinition: if there only one atom existed, there would be no bonds. However, when a second atom comes close to the first atom, the repulsive-attractive interactions happen to maintain its environmental state and the bond order at the highest value. Next, the energy of this system is distributed when the third atom comes to join. Now, the bond order decreases, causing the bonds between the first and second atoms to become weak. The same condition applies to the next new atoms. The distributed energy eventually forms a new atomic environment. From this principle, the bond order in this study is considered as a fraction of the energy distribution. It is a result of the character of the material maintaining its environment. This distribution depends on the amount of attractive energy that eventually becomes the bond.

### FEIP development

FEIP potential implements the protocol of bond order redefinition. With that protocol, this potential will translate the bond-order appropriate based on the definition of repulsive and attractive energy. Thus, there is no strict formula for FEIP as the meaning of “factory” suggests. In this study, we restricted our works to short-range interactions. Thus, we started with the short-range binding energy, the regular structure, and the strongly localised approximation of Abell’s work^27^,

1 $$E \simeq Z\left( G \right)\left( {qV_{R} \left( r \right) + h\left( {G,q} \right)V_{A} \left( r \right)} \right),$$

where *Z* is the coordination number, *q* is the net distribution of the electron, *h* is the bond order, *G* is the topology that represents the state of the atomic environment, *r* is the interatomic separation, and *V*_*A*_ and *V*_*R*_ are the attractive and repulsive energy, respectively. As noted by Abell^27^, *q* is nearly exactly equal to one in short-range approximations and bond order can thus be defined via the variational principle with respect to *r* in the following form

2 $$h\left( G \right) = \frac{{ - V_{R^{\prime}} \left( {r_{e} } \right)}}{{V_{A^{\prime}} \left( {r_{e} } \right)}},$$

where *V*_*A'*_ and *V*_*R'*_ denote the first derivative with respect to *r* of the attractive and repulsive energy, respectively. Meanwhile, *r*_*e*_ is the interatomic separation at equilibrium. Previously, the bond order was a direct function of topology *G*, as formulated by Tersoff or Brenner,

3 $$h\left( G \right) = \left( {1 + G} \right)^{ - k} ,$$

where *k* is a constant. In our redefinition, however, bond order should be a function of the fraction of the energy. Meanwhile, Eqs. (2) and (3) should be equal numerically. To solve this problem, we use the Taylor expansion of the natural logarithm, so that the relation between *G* in the right-hand side (RHS) of Eq. (3) and the energy in RHS of Eq. (2) is as follows:

4 $$G = \frac{1}{k}ln\left( {\frac{{V_{R^{\prime}} }}{{V_{A^{\prime}} }}} \right).$$

By substituting Eq. (4) into Eq. (3), the bond order for short-range interactions based on the redefinition can be written as:

5 $$h\left( G \right) = \left( {\frac{1}{k}ln\left( {\frac{{V_{R^{\prime}} \left( G \right)}}{{V_{A^{\prime}} \left( G \right)}}} \right)} \right)^{ - k} .$$

In this equation it is important is that each binding energy parameter, e.g. cohesive energy and interatomic separation equilibrium, should be a function of *G*. In practice, FEIP will use Eq. (5) to determine the bond order based on the “factory” function of repulsive and attractive energies. This means FEIP will not use the learning algorithm process, but those energy functions will instead be related to the appropriate environmental conditions.

### FEIP feature for the extended Lennard–Jones potential

Here we show the advantages of bond order redefinition for enhancing the simple empirical interatomic Lennard–Jones potential. FEIP can be used to extend the Lennard–Jones potential from two-body to many-body interactions. Here, we write the extension as

6 $$E = 4 \epsilon f\left[ {\left( \frac{A}{r} \right)^{12} - h\left( \frac{B}{r} \right)^{6} } \right],$$

where *є* is the depth of the potential well and *h* is the bond order defined according to Eq. (5), so then

7 $$h = \left( {1 + \frac{1}{k}ln\left( {\frac{1}{{r_{e}^{6} }}\left( {\frac{{A^{\prime}r_{e} - 12A}}{{B^{\prime}r_{e} - 6B}}} \right)} \right)} \right)^{ - k} .$$

The differences between the original Lennard–Jones potential and Eq. (6) are the screening functions, *f*, and *h*. Furthermore, *A*, *B*, and *r*_*e*_ are not constant parameters but are the trendline functions of *G*. Thus, the variation of the *G* topology will cause the differences in potential-well depths. These trendlines functions also will make the repulsive and attractive energy varied for every change of *G*, even *є* is constant. Currently, FEIP needs the *f* function to limit the short-range interactions. This function must be equal to one for the first nearest neighbour zone and decreases rapidly to zero for further zones.

### FEIP for carbon

In this study, we use the Morse potential to describe the universal repulsive and attractive interactions for carbon–carbon. The FEIP for carbon has the following form:

8 $$\begin{array}{*{20}l} {E = \frac{1}{2}\mathop \sum \limits_{i \ne j} f\left( {r_{ij} } \right)\left( {V_{R} \left( {r_{ij} } \right) + h_{ij} V_{A} \left( {r_{ij} } \right)} \right)} \hfill \\ {V_{R} = Aexp\left( { - \alpha \cdot \left( {r - r_{e} } \right)} \right)} \hfill \\ {V_{A} = - Bexp\left( { - \lambda \cdot \left( {r - r_{e} } \right)} \right)} \hfill \\ {h = \left( {1 + \frac{1}{k}ln\left( {\frac{B\lambda }{{A\alpha }}} \right)} \right)^{ - k} ,} \hfill \\ \end{array}$$

with

9 $$f\left( r \right) = \left\{ {\begin{array}{*{20}c} 1 & {r \le R_{1} } \\ {\frac{1}{2}\left( {1 + cos\left( {\pi \frac{{r - R_{1} }}{{R_{2} - R_{1} }}} \right)} \right)} & {R_{1} < r \le R_{2} } \\ 0 & {r > R_{2} ,} \\ \end{array} } \right.$$

where *A*, *B*, *r*_*e*_, *α* and *λ* are a function of the topology *G*. In this work, we chose the topology as follows:

10 $$\begin{array}{*{20}l} {G_{ij} = \beta^{n} \mathop \sum \limits_{k \ne i,j} f\left( {r_{ik} } \right)\gamma_{ijk} g\left( {\theta_{ijk} } \right)exp\left( {\lambda_{3}^{m} \cdot \left( {r_{ij} - r_{ik} } \right)^{m} } \right)} \\ {g\left( \theta \right) = 1 + c^{2} /d^{2} - c^{2} /\left[ {d^{2} + \left( {cos\theta_{0}- cos\theta } \right)^{2} } \right].} \\ \end{array}$$

This formula is used in the Tersoff potential. There is no standard formula for *G*. Equation (10) was chosen as the topology in order to reduce the dependency of the database, though it can reduce the accuracy of the bond order calculation due to ignoring the *π*-bonding contribution^15^. However, the most important reason to use this expression is to make comparisons with the Tersoff potential and the LCBOP as representative of short-range and long-range interactions, respectively. Thus, we can provide a clear explanation of the importance of redefining the bond order in atomic simulations.

The number of constant parameters required depends on the type of trendline functions of *A*(*G*), *B*(*G*), *r*_*e*_(*G*), *α*(*G*), and *λ*(*G*). Meanwhile, the number of databases used greatly influences these types of functions. It is not difficult to assemble the desired database from experimental or theoretical data for *A*(*G*), *B*(*G*), and *r*_*e*_(*G*). However, data for *α*(*G*) and *λ*(*G*) dominantly comes from theory. These functions represent the width of the well potential. We can determine this information with DFT or another interatomic potential which has a similar physical meaning. Several studies reveal the relationship of the exponential trend of potential energy and bond order to the coordination number^27,28^. Therefore, this study uses an exponential form for the trendline functions:

11 $$\begin{array}{*{20}c} {A\left( G \right) = A_{1} exp\left( {A_{2} G} \right),} \\ {B\left( G \right) = B_{1} exp\left( {B_{2} G} \right),} \\ {r_{e} \left( G \right) = r_{e1} exp\left( {r_{e2} G} \right)} \\ {\alpha \left( G \right) = \alpha_{1} exp\left( {\alpha_{2} G} \right),} \\ {\lambda \left( G \right) = \lambda_{1} exp\left( {\lambda_{2} G} \right).} \\ \end{array} ,$$

Uncertainty and the lack of data is another limitation in this concept. Thus, the choice of the trendline function requires physical intuition. In completing our work, this study focuses on graphite, graphene and diamond materials as the carbon allotropes for which there is abundant data. The parameters in *G* topology of Eq. (10) have a close relationship to the mechanical properties, while the others are related to the cohesive energy and lattice constant of each allotropic material type. Except for *R*_1_ and *R*_2_, we determine these parameters based on the farthest and shortest distance of the first and second nearest neighbours of the interest allotropic material, respectively. Here, we chose *R*_1_ equal to 1.8 Å (slightly over 1.55 Å, the farthest distance of the diamond first nearest neighbour), and *R*_2_ we set at 2.1 Å (below 2.46 Å, the shortest distance of the graphite second nearest neighbour), based on the experiment^29,30^. Then the standard fitting process is repeatedly carried out until the calculation results are closer to the experiment. After passing the standard fitting procedure following Tersoff’s work^11^, Table 1 shows the constant parameters suggestion for Eqs. (9)–(11).

**Table 1:** Constant parameters suggestion of FEIP for carbon.

*A*_1_ = 9.7388 eV	*k* = 0.6873
*A*_2_ = − 7.4816	*γ* = 0.3125
*B*_1_ = 19.5470 eV	*c* = 1.0000
*B*_2_ = − 6.8629	*d* = 0.9600
*r*_*e*1_ = 1.1647 Å	*m* = 3.0000
*r*_*e*2_ = 2.1665	*λ*_3_ = 0.0000
*α*_1_ = 3.6815 Å^-1^	cos *θ*_0_ = − 0.6819
*α*_2_ = 1.2167	*β* = 0.0575
*λ*_1_ = 1.8342 Å^-1^	*R*_1_ = 1.8000 Å
*λ*_2_ = 1.2852	*R*_2_ = 2.1000 Å

### Test of FEIP for carbon

In this section, we present the test results of FEIP for carbon, including the lattice constant, cohesive energy, and elastic constants. Our results are compared with experimental data and other simulations for diamond and graphene, as shown in Table 2.

**Table 2:** Comparison of the lattice constants, cohesive energies, elastic constants of diamond and graphite.

	Diamond	Graphite	Method	Ref
*a*_*lattice*_ (Å)	3.570	2.457	FEIP	
	3.567	2.459	Experimental	^29,30^
	3.645	2.492	Tersoff optimisation	^31^
	3.567	2.460	Brenner optimisation	^31^
	3.088	2.459	LCBOP	^18^
*E*_*c*_ (eV)	− 7.349	− 7.374	FEIP	
	− 7.349	− 7.374	Experimental	^30^
	− 6.537	− 7.978	Tersoff optimisation	^31^
	− 7.361	− 7.401	Brenner optimisation	^31^
	− 7.349	− 7.375	LCBOP	^18^
*C*_11_ (GPa)	902.09	1141.23	FEIP	
	1079	1109, 1440	Experimental	^32–34^
	1027	1005	ReaxFF	^35–38^
*C*_12_ (GPa)	322.21	11.51	FEIP	
	124	139	Experimental	^32,34^
	566	505	ReaxFF	^35–38^
*C*_44_ (GPa)	963.07		FEIP	
	578		Experimental	^32^
	252		ReaxFF	^35,36,38^
*C*_66_ (GPa)		564.87	FEIP	
		485, 460	Experimental	^33,34^
		186	ReaxFF	^35,38^

When compared with other simulations, FEIP gives close results to the experimental data for the lattice constant and the cohesive energy. For the elastic constants, however, FEIP gives a prediction of *C*_12_, which is far from the experimental data. Nevertheless, the results of FEIP for diamond and graphite case might be still better than other methods, even compared with the complex calculations of the best reactive force field (ReaxFF). The prediction errors resulting from FEIP for diamond and graphite are 159% and 92%, respectively. Meanwhile, ReaxFF has errors of 356% and 263%, respectively, almost twice the error of FEIP. For the elastic constants, ReaxFF has 56% error, but FEIP is 66%, except for *C*_44_, shear displacement over the basal plane of graphite.

**Figure 1:**
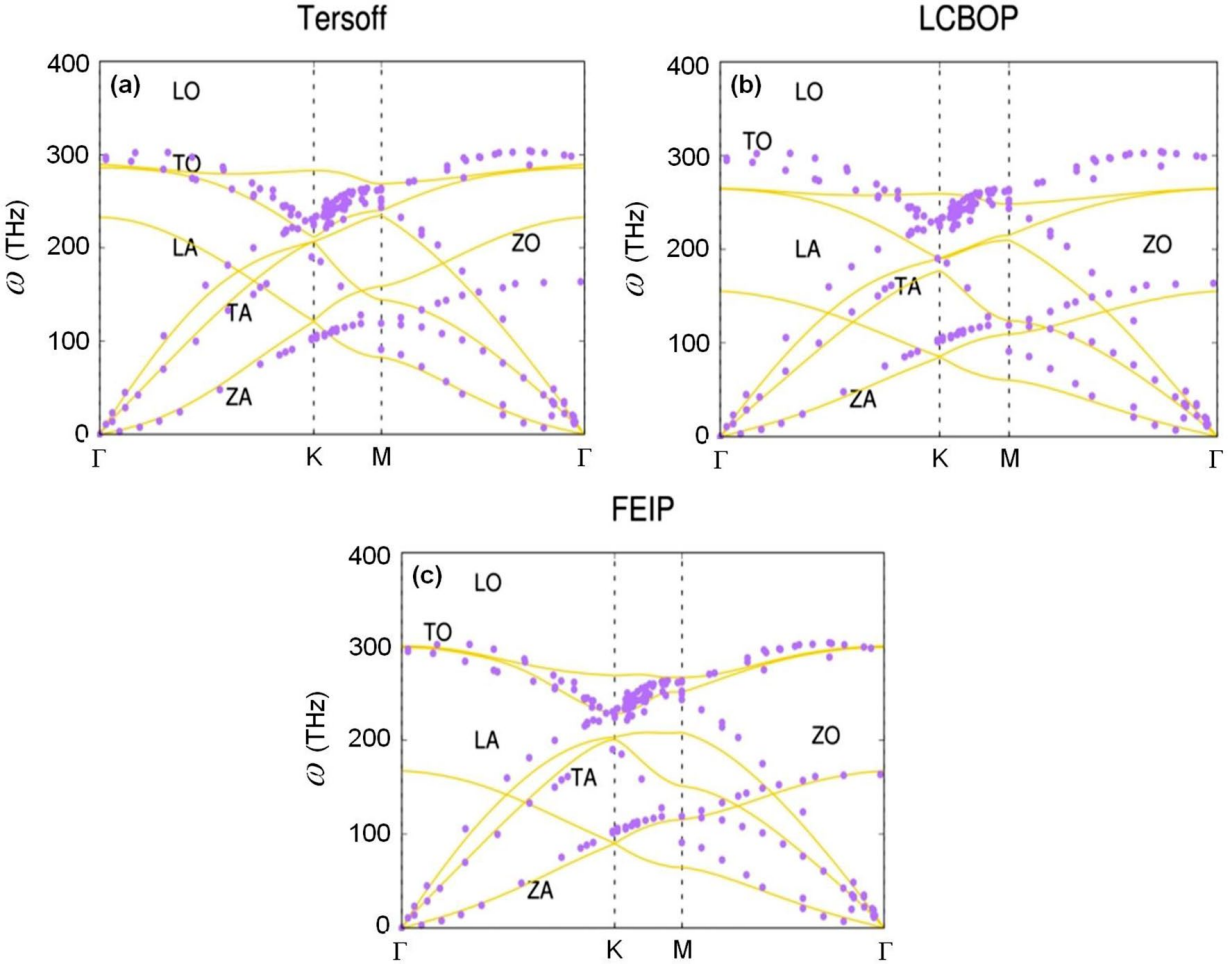
Graphene phonon dispersion calculations using (**a**) the Tersoff potential, (**b**) LCBOP, and (**c**) FEIP at 300 K. Experimental data is from Ref.^43^, except for LO and TO which were taken from Ref.^44^. Purple circle and gold solid-lines are experimental data and simulation results, respectively.

The next test is the phonon dispersion of graphene. This test is needed to verify the representation of some physical properties such as thermal and electrical conductivity^39–42^. Phonon vibration modes control the distance between atoms that affects the bond order. Therefore, the accuracy of the phonon calculation is a test of the redefinition of the bond order implemented by FEIP. For that reason, this study compares phonon calculations with LCBOP as representative of long-range interactions and the Tersoff optimisation for short-range interactions. Figure 1 shows the result.

At the Γ-point, the experimental data shows two optic mode branches, in-plane transverse (TO) and longitudinal (LO) modes at the frequency 298 THz. Meanwhile, the out-of-plane optical (ZO) mode is at 163 THz. The simulation result using the LCBOP predicts the ZO at 155 THz, while the simulation using the Tersoff potential gives a result 70 THz higher than the experimental value. Meanwhile, for the TO mode prediction, the LCBOP and Tersoff potential give results of 265 THz and 289 THz, respectively. The FEIP predicts 168 THz and 299 THz for ZO and TO mode, respectively (see Fig. 1), which is the best compared with the other potentials. Phonon path from Γ to K-point, the results using the FEIP are closer to the experimental values than the others. One notes here, at the Γ-point as the starting path, the Tersoff potential gives a non-degenerate result for the TO and LO branches, which is a contradiction with the experimental data. Next for out-of-plane accoustic (ZA) mode prediction, the results of the calculation using the FEIP for the crosses frequency of ZA/ZO due to the consequences of the point-group symmetry of graphene are 90 THz at the K-point, 12 THz lower than the experimental value. Meanwhile, the prediction based on the LCBOP is 16 THz lower. However, the Tersoff potential-based prediction is about 20 THz lower than the others.

In general, the transverse acoustic (TA) mode of the FEIP calculation is similar to that of the Tersoff potential calculations, but the LCBOP gives the closet result to the experimental data. Nevertheless, the predictions for ZO and ZA mode of the FEIP are the best. FEIP also makes a better prediction for the LO and TO modes. Especially from Γ- to K-point, the FEIP calculation results are closer to the experimental data than the others. However, the over bending mode character is not visible for all potentials.

**Figure 2:**
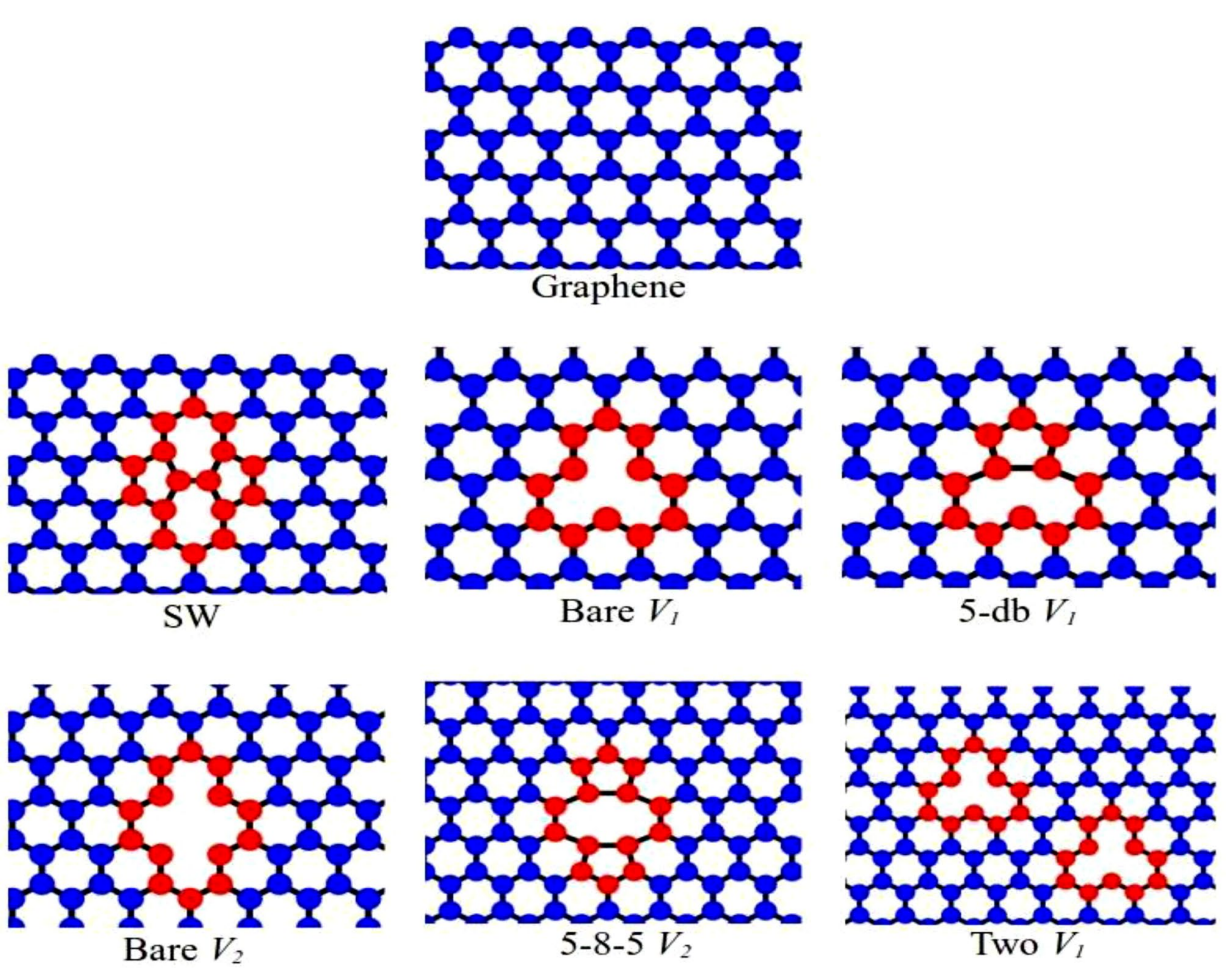
Relaxation defect structure spot of Stone–Wales (SW), bare single vacancy (*V*_1_), 5-db *V*_1_, bare (divacancy) *V*_2_, 5-8-5 *V*_2_, two *V*_1_. The top image is the graphene structure spot before defect. The blue circle and black solid line represent atoms and bond length, respectively. The red circle represents the atom that forms a defect.

The next test is the stability and accuracy of the defects system with FEIP potential. Here, we show the relaxation defect structure spot of Stone–Wales (SW), bare single vacancy (*V*_1_), pentagon dislocation reconstruction due to dangling bonds (5-db) *V*1, bare divacancy (*V*_2_), a divacancy fully sp^2^ reconstruction made of two pentagons and a central octagon (5-8-5) *V*_2_ and a divacancy with two *V*_1_ of graphene in Fig. 2. Meanwhile, the formation energy (*E*_*f*_) of vacancies and dislocation is calculated with the following formula

12 $$E_{f} = E_{d} - E_{0} - n\mu ,$$

where *E*_*d*_, μ and *E*_0_ are defect energy, the chemical potential of carbon and initial energy before defect, respectively, and n gives the number of carbon atoms that were added (*n* positive) or removed (*n* negative). We then compare their formation energy with the results of other studies presented in Table 3. As noted in the table, based on the tight-binding and DFT calculation, FEIP predicts the lowest formation energy compared to Tersoff and LCBOP for the bare vacancy.

**Table 3:** The formation energy of Stone–Wales (SW) and different types of vacancy(s) in graphene.

Defect	Formation energy (eV)						
	FEIP	Tersoff optimized ref.^31^	LCBOP ref.^18^	Airebo ref.^45^	DFT ref.^46^	Tight-binding ref.^47^	DFTB ref.^48^
Bare *V*_1_	3.06	0.52	7.59	7.55	7.6		
5-db *V*_1_	3.88	4.05	5.41	7.02	7.4		
Bare *V*_2_	4.09	0.75	10.00	10.08			
5–8-5 *V*_2_	14.4	15.7	12.9	7.35	8.7		
Two *V*_1_	6.13	1.04	15.2			14.74	
SW	− 0.25	− 3.80	− 3.94	5.13			5.9

In general, Tersoff optimized and FEIP must underestimate the result for the *V*_1_ and SW based on Airebo and DFT calculation. Meanwhile, LCBOP, which represents long-range interaction, shows the lowest prediction for SW, but FEIP has a better result. However, LCBOP is closer to the DFT calculation for bare *V*_1_ than FEIP. For the number types of divacancy and pure dislocation SW, FEIP shows better results compared to the Tersoff optimized and LCBOP, respectively. This means that FEIP of this version gives a better prediction for the case in which the number of vacancies increases and dislocation is lower. The inaccuracy result of FEIP for V1 and dislocation of graphene is due to our approaches that ignore π-bonding and short-range approximation.

**Figure 3:**
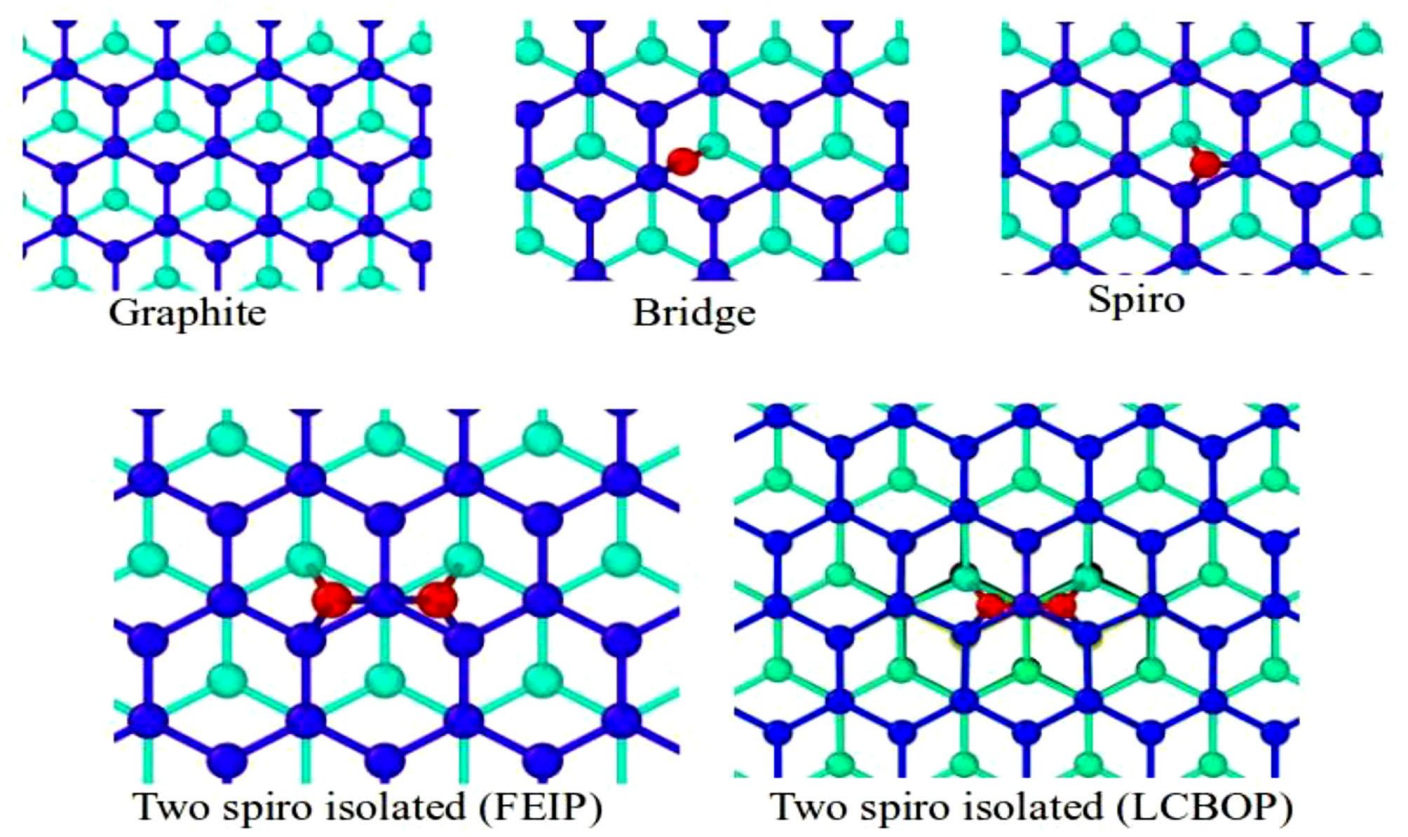
The relaxation structure spot of graphite without and with interstitial. The circle and solid line represent atom and bond length. The blue, emerald and yellow colours are the first, second and third layer from the top of graphite. The red circle(s) represent carbon interstitial between the first and second layer.

The last test for FEIP in this study is the stability and accuracy of the interstitial case in graphite. Thus, we insert one atom between the top two layers of graphite for the bridge and spiro interstitial. In that same position of the two layers, two atoms are also inserted to produce the di-interstitial of two spiro isolated. Figure 3 shows the meaning of these interstitials.

Table 4 shows the interstitial types of formation energy. From the simulation, it is known that Tersoff optimized and LCBOP failed to produce all stable interstitial structures (not shown on the figure), except for the two spiro isolated of LCBOP. Meanwhile, calculations using FEIP show the opposite result.

**Table 4:** The formation energy of interstitial types in graphite.

Interstitial	Method	Formation energy (eV)	Refs.
Bridge	FEIP	5.96	
	DFT	8	^49^
	DFT	7.68	^50^
Spiro	FEIP	6.65	
	DFT	6.5	^49^
	DFT	6.2	^50^
Two spiro isolated	FEIP	13.4	
	LCBOP	13.4	^18^
	DFT	10.23	^51^

FEIP results in a close prediction to DFT calculation for spiro. Meanwhile, for bridge and two spiro isolated interstitials show a difference of almost 3.0 eV. These happen because FEIP in this version ignores the π-bonding; as a result, the carbon interstitial gets even radial distribution energy from the top and bottom layers. This explains why the carbon for the bridge interstitial in Fig. 3 binds to the two closest atoms of the two layers that flank it, which contradicts the DFT result^50^.

## Discussions

### Extended Lennard–Jones potential

The Lennard–Jones potential is generally used for rare gas interactions and simple fluids^52–55^ In general, particles interacting following this potential are stable in the hexagonal close-packed (hcp) structure at zero temperature and pressure^55–57^, but some rare gases such as Ne and Ar form face-centred cubic (fcc) structures^58,59^. To anticipate this rare gas solid (RGS) problem, many researchers modify this potential to predict the onset of crystallisation in supercooling, surface tension, critical nucleus size and nucleation ratse^60,61^. Schwerdtfeger et al.^62^ extended the Lennard–Jones potential by using two-body potentials to improve structure calculations of rare gas clusters and the solid state. They argue that the use of the many-body expansion does not change the preference for hcp over fcc due to zero-point vibrational effects. However, that statement disagrees with the Lennard–Jones Embedded-Atom (LJEA) potential from the work of Baskes^63^. This potential embeds the energy of each atom into the background electron density to allow investigation of many-body effects. The LJEA potential is used to calculate properties of an fcc material such as elastic constants, Bain transformation and defect properties as a function of many-body parameters. The result of the calculations sthat hows the ground state structure includes all phases; meanwhile, the melting point of fcc structures decreases while the many-body interactions increase.

We argue that the hcp preference of the Lennard–Jones-based calculations is due to the inability of this potential to adjust the energy requirements to the state of the atomic environment. In the LJEA potential, each atom is embedded in the background electron density provided by neighbouring atoms. Thus, the LJEA potential takes the energy as a summation of the pair interactions and the embedding energy as a function of the local background electron density. However, the LJEA potential assumes that the electron cloud around each atom is spherical, which makes this potential good for the fcc structure. The FEIP differs in that there is no pair interaction summation since every attractive and repulsive energy is a function of topology. FEIP will predict the Lennard–Jones energy requirement associated with the current environment topology. In this way, the FEIP offers a calculation which adapts to every possible crystal structure. However, here we focus on presenting the Lennard–Jones extension as a FEIP feature. To show the bond order requirements via FEIP, we chose the carbon–carbon interaction to test the accuracy because it is more complex than the rare gas case.

### FEIP for carbon: bond order redefinition reason

Although FEIP is more accurate in predicting lattice constants and cohesive energy, the need for bond order redefinition begins with the accuracy of the elastic constants. Changing the position of each atom due to strain and tension will cause changes to the bond order. Thus, the accuracy of these properties is evidence in favour of redefining the bond order. The previous study corroborates this statement that the elastic properties come from many-body interactions that are counted by the bond order^43^.

ReaxFF uses its own definition for a complex bond order calculation summed from *σ*, *π*, and *π*–*π* bonding, however, the results in Table 2 show that calculations of *C*_12_ and *C*_44_ fall too far from the experiment. Although this current FEIP uses short-range and σ-bonding, with redefinition, this work shows better results. Especially for *C*_66_, ReaxFF has underestimated the value compared with FEIP. In addition to our ignoring the π-bonding approach in the derivative FEIP formula, another cause of overprediction for these elastic constants is the lack of carbon allotroph data so that the absolute values of the *A*_2_ and *B*_2_ parameters are relatively large; this means a small change (0.33%) in *G* topology will result in *A*(*G*) and *B*(*G*) shifting up to 7 percent. Thus, the FEIP result confirms the accuracy of the elastic constant due to the concept of bond order. Thus, comparing the elastic constants is our chance to prove the needs redefinition.

We analysed the calculation of phonon dispersion to better understand the need for a redefinition of the bond order. The accuracy of phonon modes presented by FEIP, especially at low frequency, is due to the different character of this potential compared with LCBOP and the Tersoff potential. For the longitudinal accoustic (LA) mode, however, FEIP gives a result similar to a calculation using a force constant and valence force field (VFF) methods in which the fourth nearest-neighbour interactions are considered^64,65^, except there is no tangent frequency with LO mode at the K-point. Thus, the weakness of FEIP in predicting phonon modes at high frequencies compared to experimental data is due to the short-range interaction approximation, not the bond order redefinition. This means that the LO and TO modes are the causes of the long-range interactions.

**Figure 4:**
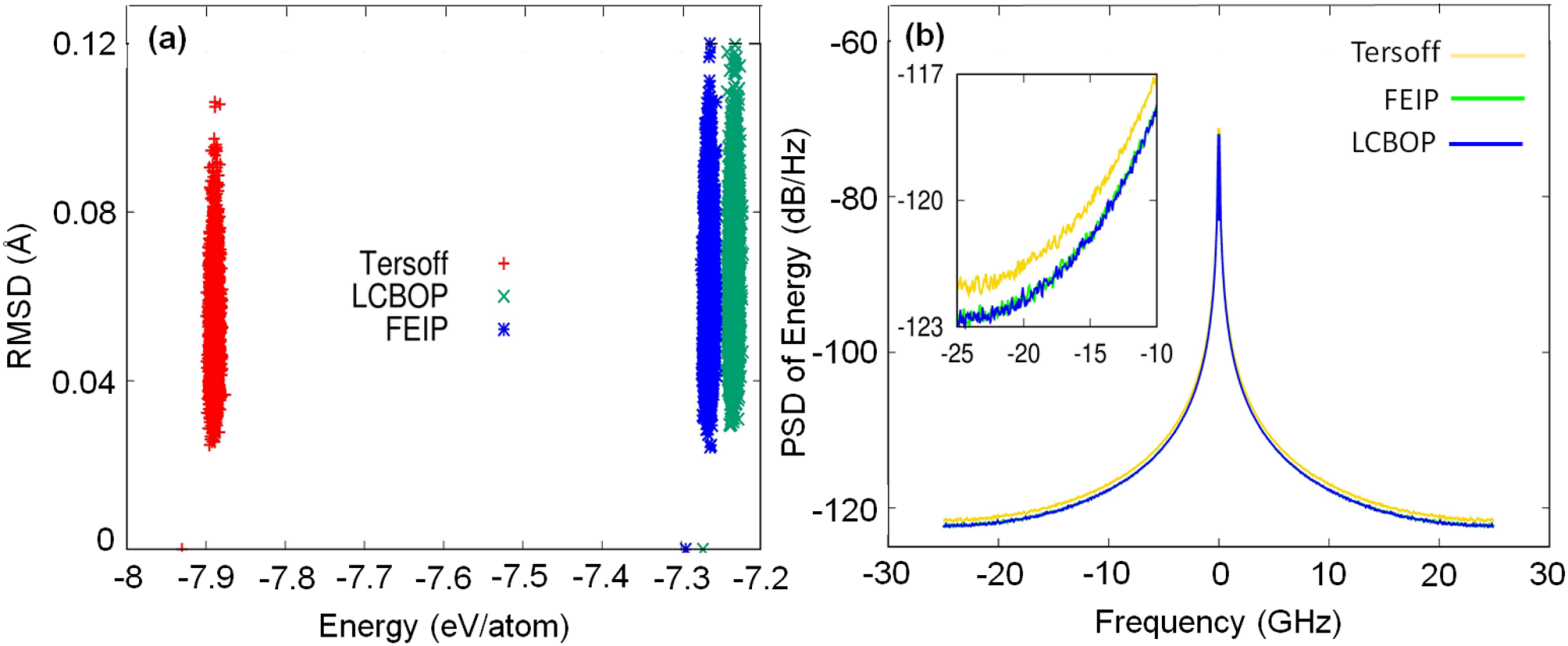
(**a**) The plot of RMSD with respect to the energy of all potentials used during phonon computation. (**b**) Power spectral density (PSD) of the energy extracted during the phonon calculation process.

To see how the bond order redefinition affects the accuracy of the simulation, we studied the carbon atoms during the phonon calculations. For that reason, a plot of root mean square deviation (RMSD) with respect to the total energy is shown in Fig. 4a. This study takes one particular atom of graphene juxtaposed with its nine nearest neighbour atoms to calculate the RMSD. From Fig. 4a, it is clear that the Tersoff potential gives a lower energy prediction than the others because the carbon atoms cannot move freely and are localised. However, LCBOP and FEIP show different conditions. Both potentials give an energy higher than the Tersoff potential because the carbon atoms move freely and provide more available phonon modes. FEIP provides energy predictions which almost coincide with the LCBOP calculations, where the LCBOP prediction is slightly higher. This highest-energy state of the LCBOP causes the atoms to move too freely, making the predictions for high frequency phonon modes inaccurate. The FEIP applies a short-range interaction approach while the LCBOP uses a long-range interaction approach. However, with the concept of bond order redefinition, the FEIP can adjust the energy requirements to the state of its atomic environment.

Figure 4b shows the power spectral density (PSD) of energy, which reveals the cause of the inability of the Tersoff potential to adjust its energy requirements to the environment. We can see that the magnitude and amplitude of the white noise from the Tersoff are higher than in the FEIP and the LCBOP. Outside of the range of frequency from − 20 to 20 GHz, the level of noise fluctuation becomes more significant in the Tersoff potential, meaning that up to 500 ps (1/20 GHz) is not sufficient to reduce the noise. This situation ultimately results in a bond that is too strong compared to other potentials. The FEIP shows a different result during the phonon calculation process, in that the magnitude and amplitude of white noise are similar to the results from the LCBOP. Thus, the bond order redefinition described in this study can reduce simulation noise so that convergence is easy to achieve even though it does not apply the long-range interaction approach. Another advance of implementing this redefinition is the improvement in calculation speed. Although the Tersoff potential is faster than FEIP, Table 5 reveals that to give accuracy similar to the long-range interactions of LCBOP, FEIP needs 63.28 min, which is nearly three times faster than LCBOP.

**Table 5:** Phonon dispersion simulation speed using the Tersoff potential, LCBOP, and FEIP.

Methods	Duration (min)	TO (THz)	LO (THz)	LA (THz)	ZO (THz)	ZA (THz)
Experiment		Г-point: 298	Г-point: 298 K-point: 231	K-point: 231	Г-point: 163 K-point: 102	K-point: 102
LCBOP	166.95	Г-point: 265	Г-point: 265		Г-point: 155 K-point: 86	K-point: 86
Tersoff	12.38	Г-point: 289	Г-point: 233		Г-point: 233 K-point: 122	K-point: 122
FEIP	63.28	Г-point: 301	Г-point: 301		Г-point: 168 K-point: 90	K-point: 90

## Methods

In this study, all simulations were run using the open-source code Large-scale Atomic/Molecular Massively Parallel Simulator (LAMMPS)^66^. Meanwhile, the open-source code Octave (GNU) was used to analyse the spectrum^67^. To redefine the bond order, we focused on carbon–carbon interactions in which graphene and diamond were selected as materials. The standard fitting procedure was used to determine the parameters of the FEIP. Meanwhile, for testing the mechanical properties, this study applies the solid-continuum methodology^68^.

This study also used a graphene single-layer and graphite four-layer system with 2508 and 4032 carbon atoms, respectively, for defects testing. We then deleted one or more carbons to represent the vacancy and dislocation. Meanwhile, one and two carbons were added between layers of the graphite system to test the carbon interstitial. After that, we compared the FEIP result testing to the Tersoff and LCBOP potential for accuracy and stability of the defects system with relaxation method.

For phonon testing, we use Kong’s methodology, which is included in LAMMPS^69,70^. A hexagonal graphene system with 200 atoms was selected for this study. The system was allowed to remain in equilibrium at 300 K for six million iterations with an NVT ensemble. During the equilibrium process, the simulation ran with a two-femtosecond timestep. The phonon distribution was calculated directly after one million iterations. The FEIP was compared to the Tersoff potential and the LCBOP as a representation of short-range and long-range interactions, respectively.

## Conclusions

We have constructed the bond order redefinition, treating it as a fraction of the energy distribution. This distribution depends on the amount of attractive energy present, which eventually becomes the bond. We developed the factory empirical interatomic potential (FEIP) that implements the bond order redefinition and uses a short-range interaction approach. From the calculation for the elastic constants, the lattice constant and the cohesive energy of graphite and diamond, the FEIP gives a close result to the experimental data, better than the complex reactive ReaxFF potential for these carbon allotropes. This study also conducts the stability and accuracy tests on defects for graphene and graphite. As a result, FEIP can provide good predictions for large vacancies and dislocations. In the case of carbon interstitial in graphite, FEIP can produce stable defects but not with Tersoff and LCBOP. Except for the two spiro isolated, LCBOP and FEIP provide the same energy predictions and are close to the DFT calculations. However, because the FEIP for this version ignores π-bonding, the defect structure for the bridge is different from the DFT view, where the FEIP results show that the interstitial carbon binds to the two nearest neighbour atoms from the graphite layer flanking it. We compared the calculations for graphene phonon dispersion using FEIP, the Tersoff potential to represent short-range interactions, and the LCBOP to represent long-range interactions. The results show that even the FEIP uses short-range interactions for carbon, but the accuracy is similar to LCBOP due to the inherent ability of FEIP to reduce the noise. Moreover, FEIP performs the calculation almost twice as fast as the LCBOP. However, the need for abundant data for different types of allotropes will influence the accuracy of the calculation, especially for elastic constants. Another advantage of implementing the redefinition is that FEIP can transform the simple two-body potential into a many-body interaction that is useful to enhance the accuracy of potentials such as the Lennard–Jones. Thus, we need the bond order redefinition for better accuracy and faster simulations, which is useful in the future study of atomic mechanisms.

## Data Availability

No datasets were generated or analysed during the current study.
